# Antioxidant Properties of a *Rhodiola rosea* Constituent, Rosavin

**DOI:** 10.3390/life16091510

**Published:** 2026-09-10

**Authors:** Drew F. Brink, Tiara L. Sapp, Trwska S. Ghafoor, Paige A. Boyland, Yukako C. Tamazawa, Guneet Kaur, Nataliia V. Shults, Robert D. Sullivan, Yuichiro J. Suzuki

**Affiliations:** 1 Department of Biochemistry and Molecular & Cellular Biology, Georgetown University Medical Center, Washington, DC 20057, USA; 2 Department of Pharmacology and Physiology, Georgetown University Medical Center, Washington, DC 20057, USA; 3 Department of Biology, Georgetown University, Washington, DC 20057, USA; 4 Capitol Anesthesia Associates, Frederick, MD 21701, USA

**Keywords:** antioxidant, hydrogen peroxide, oxidative stress, *Rhodiola rosea*, reactive oxygen species, rosavin, salidroside

## Abstract

*Rhodiola rosea* is a traditional medicinal plant with reported effects in supporting the body’s response to physical, environmental, and emotional stressors. The present study investigated the antioxidant properties of *Rhodiola rosea* chemical constituents to provide insight into their potential mechanisms of action. Using in vitro biochemical assays, we showed that a primary chemical component of *Rhodiola rosea*, rosavin, significantly decreased hydrogen peroxide (H_2_O_2_) levels dose-dependently, as determined using multiple H_2_O_2_ assays. Structural analysis revealed that the intact phenylpropanoid glycoside architecture of rosavin is required for activity, as its individual components, arabinose, rosin and cinnamyl alcohol, showed no inhibitory effect. Further investigation demonstrated that rosavin attenuates H_2_O_2_-mediated oxidation of thiol groups, supporting a role in cellular redox regulation. In cultured human cells, rosavin significantly mitigated reductions in cell viability induced by H_2_O_2_ exposure, indicating cytoprotective effects under oxidative stress conditions. Collectively, these findings identify rosavin as a structurally dependent antioxidant component of *Rhodiola rosea* that modulates H_2_O_2_-associated oxidative stress.

## 1. Introduction

*Rhodiola rosea*, commonly known as “roseroot” or “golden root,” is a perennial flowering plant in the Crassulaceae family whose medicinal properties are derived from its root [1,2,3,4,5,6,7]. *Rhodiola* species have been historically used to enhance resilience to physical and psychological stress and to treat fatigue, infection-related weakness, and chronic illness [1,6,8]. Reported pharmacological effects include anti-inflammatory, antioxidant, antidepressant, antimicrobial, immunomodulatory, and cardio-, neuro-, and hepatoprotective activities, along with central nervous system stimulation and enhanced work capacity [1,7,9]. *Rhodiola rosea* grows in high-altitude, subarctic environments characterized by low temperatures and harsh environmental conditions, including mountainous regions throughout Europe, Asia, and North America [1,4,5,6,7,8,9]. The medicinal properties of *Rhodiola rosea* are primarily attributed to its diverse group of bioactive constituents, including phenolic glycosides, polyphenols, organic acids, and essential oils [1,3,5,7]. The principal compounds associated with its effects are the phenylethanoids and phenylpropanoids, such as salidroside, tyrosol, rosavin, rosarin, rhodionin, rhodiosin, and rosin [1,3]. Commercial preparations are typically standardized to 1% salidroside and 3% rosavin; however, the content of these active compounds varies widely due to environmental and processing factors such as geographic origin, climate, harvest conditions, and extraction or drying methods, contributing to batch-to-batch variability and potential differences in therapeutic effects [4].

Salidroside, *p*-hydroxyphenylethyl-*O*-*β*-D-glucopyranoside, a phenylethanoid glycoside, consists of a tyrosol aglycone linked to a glucose moiety [2,5]. It is widely distributed across *Rhodiola* species and is commonly used as a chemical marker for species identification [2,4]. The extent to which salidroside directly contributes to antioxidant capacity, particularly relative to other *Rhodiola* constituents, remains incompletely understood.

Phenylpropanoids such as rosavin, rosarin, and rosin are characteristic constituents of *Rhodiola rosea* and *Rhodiola sachalinensis*, with rosavin representing the major phenylpropanoid constituent [4]. Rosavin, cinnamyl-(6′-*O*-*α*-L-arabinopyranosyl)-*O*-*β*-D-glucopyranoside, is a cinnamyl-alcohol derivative containing a disaccharide composed of glucose and arabinose [5]. It has been associated with antioxidant, lipid-lowering, analgesic, antiradiation, antitumor, and immunomodulatory effects [7]. Structurally, rosavin lacks a free aromatic hydroxyl group commonly present in classical para-hydroxylated phenolic antioxidants; however, its phenylpropanoid backbone contains an extended conjugated system that permits electron delocalization, potentially enabling modest radical stabilization [9]. The disaccharide moiety may further influence redox behavior, as glycosidic substitution generally diminishes the capacity of hydroxyl groups to directly participate in redox chemistry [10]. Both rosavin and salidroside have been extensively studied for their neuroprotective, anti-inflammatory, and antioxidant activities [1,9,11]. However, it remains unclear how much of this effect arises from direct radical scavenging.

The present study investigated the antioxidant properties of the *Rhodiola rosea* constituents to provide mechanistic insight into their biological activity. We provide evidence from multiple assays for rosavin’s ability to directly scavenge H_2_O_2_.

## 2. Materials and Methods

### 2.1. Chemicals

*Rhodiola* Root Standardized Fluid Extract (Alcohol-Free) that contains *Rhodiola rosea* L. Root Extract (Standardized for 3 mg Rosavins and 1 mg Salidrosides per mL serving of liquid extract) was obtained from Nature’s Answer (Hauppauge, NY, USA). Rosavin (Cat# A424145; Cas No. 84954-92-7; purity 99+%) and salidroside (Cat# A130275; Cas No. 10338-51-9; purity 98%) were purchased from Ambeed (Buffalo Grove, IL, USA). Arabinose (Cat# T13752; Cas No. 5328-37-0; purity 99.94%), rosin (Cat# T4S0398; Cas No. 85026-55-7; purity 99.85%), cinnamyl alcohol (Cat# T5S1550; Cas No. 104-54-1; purity 99%), and rosavin (Cat# T3886; Cas No. 84954-92-7; purity 99.99%) were purchased from TargetMol (Wellesley Hills, MA, USA). Catalase from bovine liver (Cat# C-9322), 5,5′-dithiobis (2-nitrobenzoic acid) (DTNB; Cat# D8130), and L-cysteine (Cat# C-7755) were from Sigma-Aldrich (St. Louis, MO, USA).

### 2.2. Hydrogen Peroxide (H_2_O_2_) Detection Assays

Multiple assays were used to detect H_2_O_2_ to strengthen the conclusions.

An EpiQuik In-Situ and Ex-Situ Hydrogen Peroxide Assay Kit (Cat# P-6005) was purchased from EpigenTek (Farmingdale, NY, USA). The fluorescence signals produced through the oxidation of a fluorogen, 10-Acetyl-3,7-dihydroxy-phenoxazine (Amplex Red), by H_2_O_2_ were measured using a POLARstar OPTIMA Fluorescence Microplate Reader (BMG Labtech, Cary, NC, USA). The *Rhodiola rosea* extract alone did not affect this fluorescence-based H_2_O_2_ assay; however, the extract’s intrinsic brown color did not allow assessment by colorimetric assays. Thus, this assay was used for experiments involving the extract.

An Amplite Colorimetric Hydrogen Peroxide Assay Kit (Cat# 11500) was purchased from AAT Bioquest (Pleasanton, CA, USA). The oxidation product of Amplite IR, exhibiting an intense blue color, was measured at an absorbance of 650 nm using an EMax Plus Microplate Reader (Molecular Devices, LLC., San Jose, CA, USA).

Insta-Test Peroxide Test Strips Low Range (Cat# 2984LR) were purchased from LaMotte (Chestertown, MD, USA). Test strips were inserted into the reaction mixtures for 1 min at room temperature and photographed. 

H_2_O_2_ levels were also assessed by detecting the fluorescence of the Europium–Tetracycline–H_2_O_2_ complex [12,13] at an excitation wavelength of 390 nm and emission wavelength of 620 nm using a POLARstar OPTIMA Fluorescence Microplate Reader.

H_2_O_2_ levels in cell culture medium samples were measured using the QuantiChrom Peroxide Assay Kit (Cat# DIOX-250) purchased from BioAssay Systems (Hayward, CA, USA) on an EMax Plus Microplate Reader.

### 2.3. Superoxide Detection Assay

Superoxide anion radicals generated by the xanthine/xanthine oxidase system were measured using the Amplite Colorimetric Superoxide Dismutase (SOD) Assay Kit (Cat# 11305) purchased from AAT BioQuest. A colored product generated from ReadiView SOD560 upon reaction with superoxide was monitored at a wavelength of 562 nm using an EMax Plus Microplate Reader.

### 2.4. 5,5′-Dithiobis-(2-nitrobenzoic acid) (DTNB) Assay

Oxidation of the sulfhydryl group of L-cysteine was monitored using DTNB (Ellman’s Reagent) as previously described [14,15]. DTNB reacts with free sulfhydryl groups to produce 2-nitro-5-thiobenzoate (TNB), which is a yellow-colored product quantified at an absorbance of 412 nm using an EMax Plus Microplate Reader.

### 2.5. Cell Culture Experiments

Human pulmonary artery smooth muscle cells (Catalog #3110) were purchased from ScienCell Research Laboratories (Carlsbad, CA, USA) and cultured in Smooth Muscle Cell Medium (Cat #1101) at 37 °C in 5% CO_2_ in a humidified incubator. We used passage numbers 3–6 for experiments. Viable cells were assessed using the Cell Counting Kit-8 (CCK8; Dojindo Molecular Technologies, Rockville, MD, USA). After plating at 5000 cells/well in a 96-well plate and pre-incubating for 24 h, cells were treated with H_2_O_2_ or rosavin dissolved in water.

### 2.6. Statistical Analysis

Means and standard errors of the mean (SEMs) were computed. Comparisons between two groups were analyzed using a two-tailed Student’s t-test, and comparisons between more than two groups were analyzed using one-way analysis of variance (ANOVA) followed by Tukey HSD test using the Statistics Kingdom software (statskingdom.com). Continuous variables were assessed for the assumptions required for parametric testing. Data independence was ensured through the study design and independent sampling. Normality of the data was evaluated using the Shapiro–Wilk test at a significance threshold of α = 0.05. The assumption of normality was met (*p* = 0.3701), showing no statistically significant departure from a normal distribution. Homogeneity of variances was verified using Levene’s test. *p* < 0.05 was considered statistically significant.

## 3. Results

### 3.1. Rhodiola rosea Inhibits H_2_O_2_

**Figure 1:**
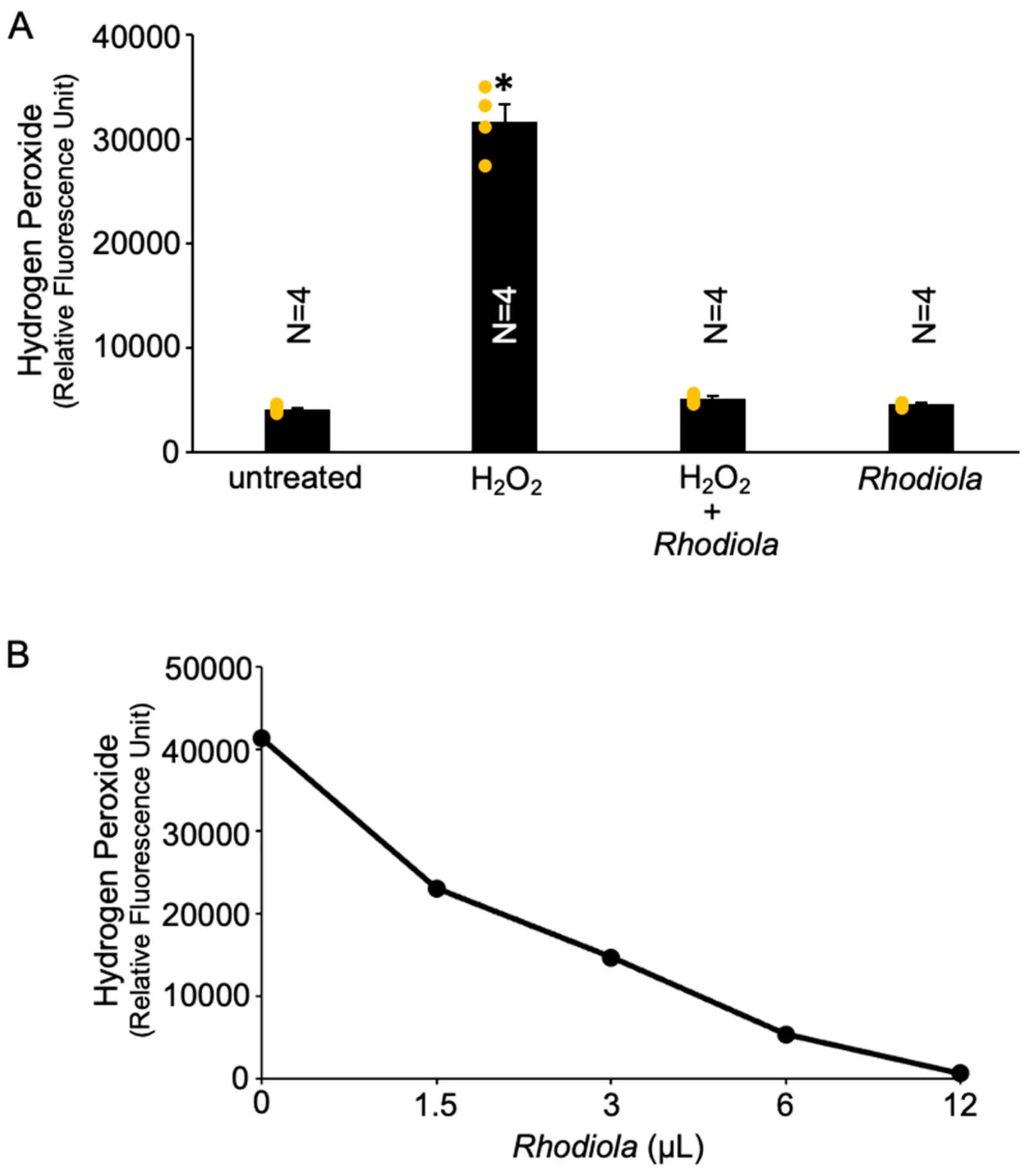
Effects of *Rhodiola rosea* on H_2_O_2_. (**A**) EpiQuik In-Situ and Ex-Situ Hydrogen Peroxide (H_2_O_2_) Assay (EpigenTek) was used to determine the effects of the *Rhodiola rosea* extract on H_2_O_2_. The assay reactions contained a 4 μM final concentration of H_2_O_2_ along with 6 μL Nature’s Answer *Rhodiola* Root Standardized Fluid Extract. The bar graph represents the mean ± SEM. * indicates that the value is significantly different from other groups at *p* < 0.05 as determined by one-way ANOVA. (**B**) Dose–response effects of various doses of this *Rhodiola* extract on a 2.5 μM final concentration of H_2_O_2_.

To characterize the antioxidant properties of *Rhodiola rosea* and its components, we tested the effects of Nature’s Answer *Rhodiola* Root Standardized Fluid Extract, a widely used product, on H_2_O_2_ levels using a 10-acetyl-3,7-dihydroxy-phenoxazine fluorogen-based EpiQuik H_2_O_2_ Assay. As shown in Figure 1A, the addition of H_2_O_2_ to the assay increased the fluorescence signal. In the presence of *Rhodiola rosea* extract, the H_2_O_2_-associated fluorescence signal was attenuated, suggesting that the extract reduces H_2_O_2_ levels. This effect of *Rhodiola rosea* was dose-dependent (Figure 1B).

### 3.2. Rosavin Decreases H_2_O_2_

**Figure 2:**
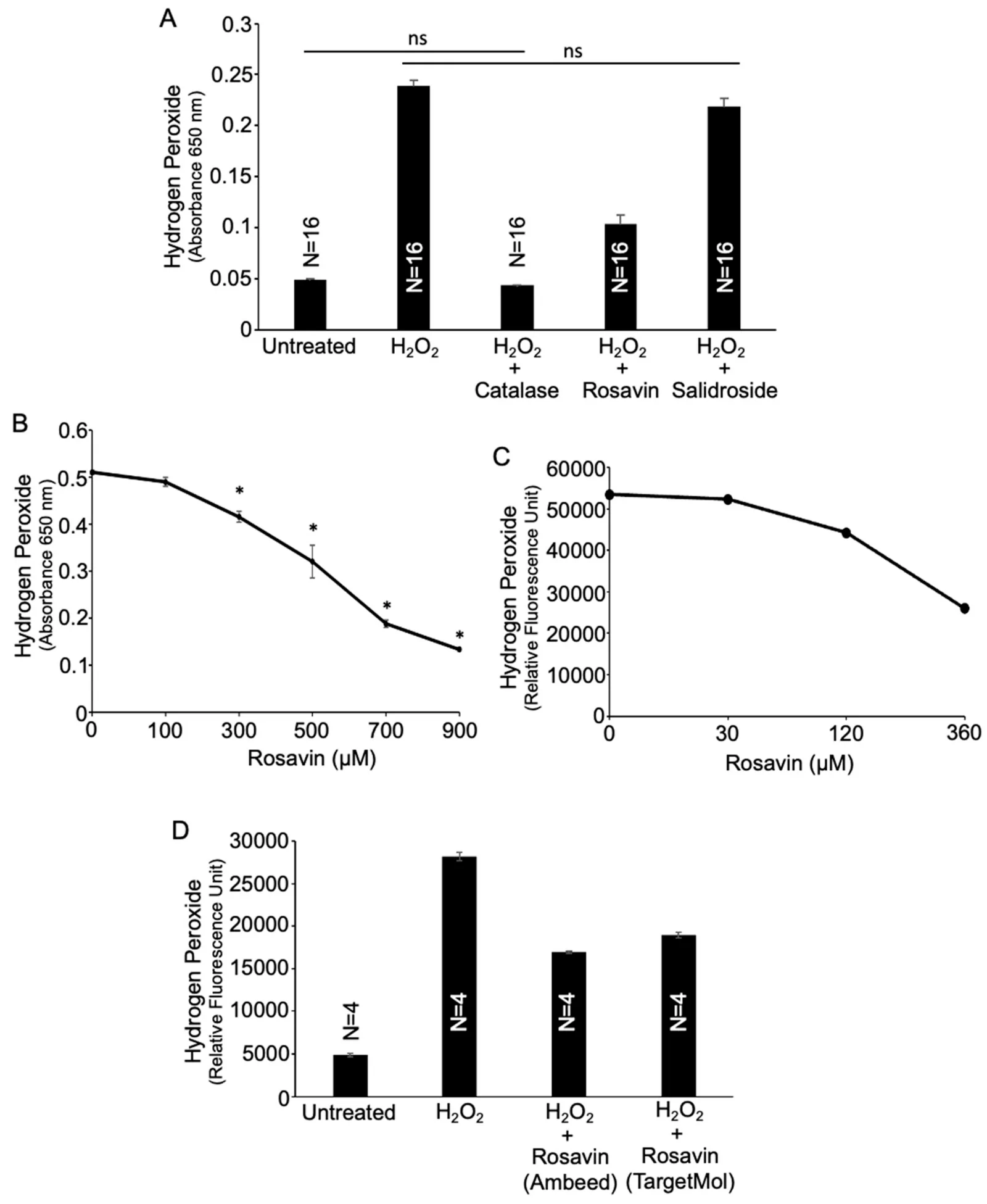
Rosavin decreases H_2_O_2_. (**A**) The Amplite Colorimetric Hydrogen Peroxide Assay (AAT Bioquest) was used to determine the effects of rosavin and salidroside on H_2_O_2_. The assay reactions contained a final concentration of 10 μM H_2_O_2_, catalase (1000 U/mL), a known H_2_O_2_ scavenger, rosavin (400 μM), and/or salidroside (400 μM). The bar graph represents the mean ± SEM. “ns” indicates no significant difference between groups. All values without an “ns” designation are significantly different at *p* < 0.05 as determined by one-way ANOVA. (**B**) Rosavin produced a concentration-dependent decrease in the H_2_O_2_ signal, as determined by the Amplite Colorimetric Hydrogen Peroxide Assay. The assay reactions contained a final concentration of 10 μM H_2_O_2_ and various concentrations of rosavin. The line graph represents the mean ± SEM (n = 4). * denotes groups that are significantly different from the 0 μM rosavin control at *p* < 0.05 as determined by one-way ANOVA. (**C**) Rosavin produced a concentration-dependent decrease in the H_2_O_2_ signal, as determined by the EpiQuik In-Situ and Ex-Situ Hydrogen Peroxide Assay. The assay reactions contained a final concentration of 4 μM H_2_O_2_ along with various concentrations of rosavin. (**D**) The EpiQuik In-Situ and Ex-Situ Hydrogen Peroxide Assay showed that the effects of rosavin obtained from Ambeed, as shown above, were reproduced using rosavin from another source (TargetMol). All the values are significantly different from each other at *p* < 0.05. However, *p* values for comparisons between the “H_2_O_2_” and “H_2_O_2_ + rosavin” groups ranged from *p* = 5.4 × 10^−10^ to 3.1 × 10^−11^, whereas the *p*-value for the comparison between “Ambeed” and “TargetMol” groups was substantially larger at *p* = 0.004. Thus, the small difference in rosavin effects between the two sources is likely due to minor batch effects, rather than a discrepancy.

**Figure 3:**
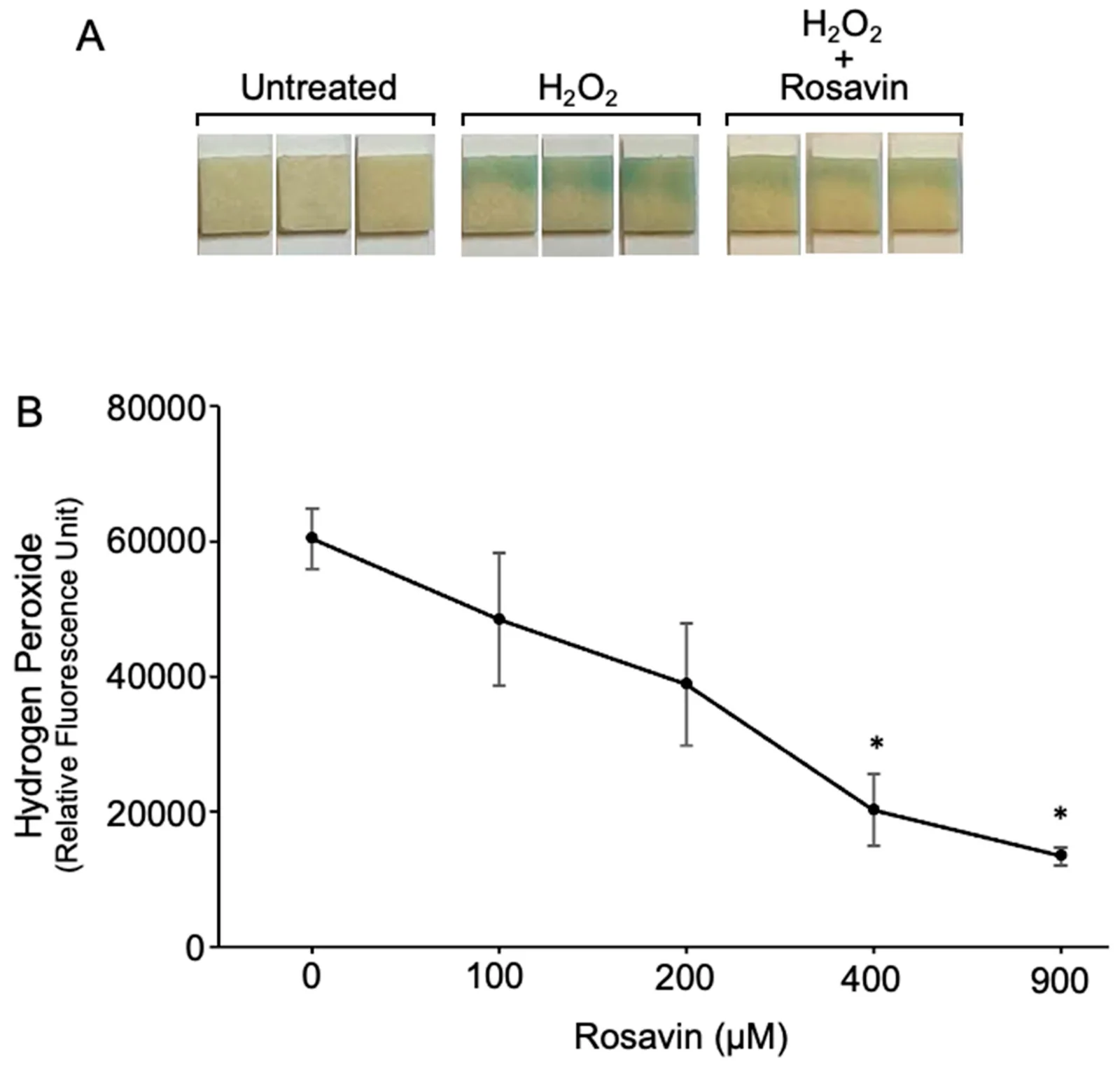
Rosavin decreases H_2_O_2_ levels, as assessed using peroxide strips and the Europium–Tetracycline assay. (**A**) Reaction mixtures contained a final concentration of 10 μM H_2_O_2_ and 400 μM rosavin. Insta-Test Peroxide Test Strips, Low Range (LaMotte, Chestertown, MD, USA) were inserted into the reaction mixtures for 1 min at room temperature. Representative results from the peroxide strips are shown. (**B**) The Europium–Tetracycline assay was used to determine the effects of rosavin on H_2_O_2_ levels. The line graph represents the mean ± SEM (n = 5). * Indicates that the value is significantly different from the 0 μM rosavin control at *p* < 0.05 as determined by one-way ANOVA.

Given that the major constituents of *Rhodiola rosea* are rosavin and salidroside, the effects of these compounds on H_2_O_2_ levels were evaluated. Initial experiments using the Amplite Colorimetric H_2_O_2_ Assay showed that rosavin alone did not interfere with assay readout. Figure 2A demonstrates that the H_2_O_2_ signal was significantly attenuated by catalase (a known H_2_O_2_ scavenger that should serve as a positive control at the concentration used). Rosavin also significantly reduced H_2_O_2_, but salidroside did not show a significant effect at the same rosavin concentration (N = 16). The inhibitory effects of rosavin on H_2_O_2_ were found to be dose-dependent as determined by both the absorbance Amplite Assay (Figure 2B) and the fluorescence-based EpiQuik Assay (Figure 2C). In this study, rosavin purchased from Ambeed was used for all the experiments, except Figure 2D, which shows that rosavin from another source (TargetMol) also affected H_2_O_2_. Consistent results on the inhibitory effects of rosavin were also obtained using other detection methods, including the LaMotte Insta-Test Hydrogen Peroxide Test Strips (Figure 3A) and the Europium-Tetracycline assay (Figure 3B).

**Figure 4:**
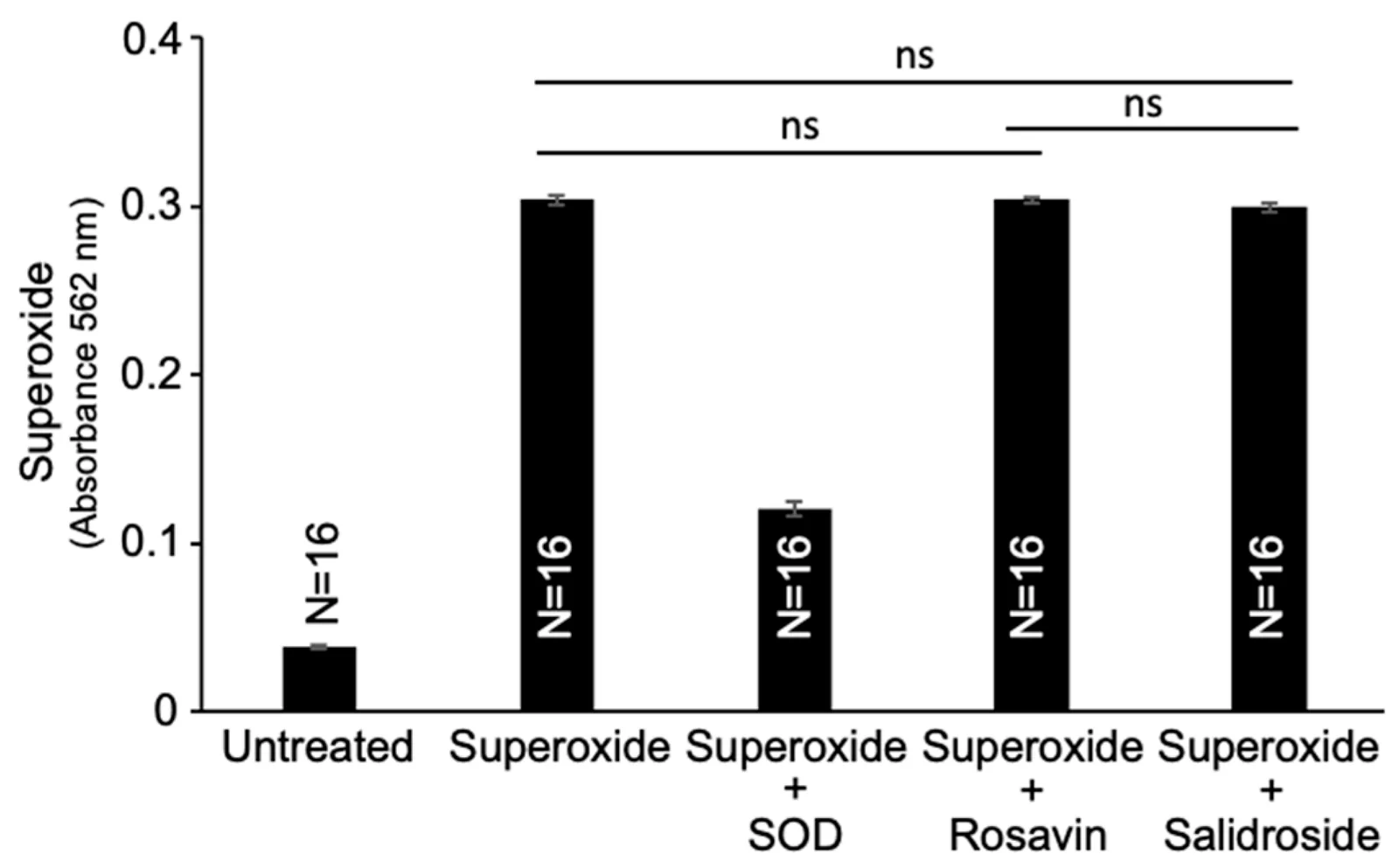
Neither rosavin nor salidroside affected the superoxide anion radical under the tested assay conditions. The Amplite Colorimetric Superoxide Dismutase (SOD) Assay (AAT Bioquest) was used to determine the effects of rosavin and salidroside on superoxide produced by xanthine plus xanthine oxidase. Superoxide was detected using ReadiView SOD560. The assay reactions contained xanthine plus xanthine oxidase, SOD (5 U/mL), a known superoxide scavenger, rosavin (400 μM), and/or salidroside (400 μM). The bar graph represents the mean ± SEM. ns indicates no significant difference between groups. Values without an “ns” designation are significantly different at *p* < 0.05, as determined by one-way ANOVA.

By contrast, rosavin and salidroside did not influence the superoxide anion radical levels, as monitored using the Amplite Colorimetric Superoxide Assay. While the positive control, superoxide dismutase (SOD), effectively decreased the superoxide signal, neither rosavin nor salidroside exerted any effects under the assay conditions tested (Figure 4).

**Figure 5:**
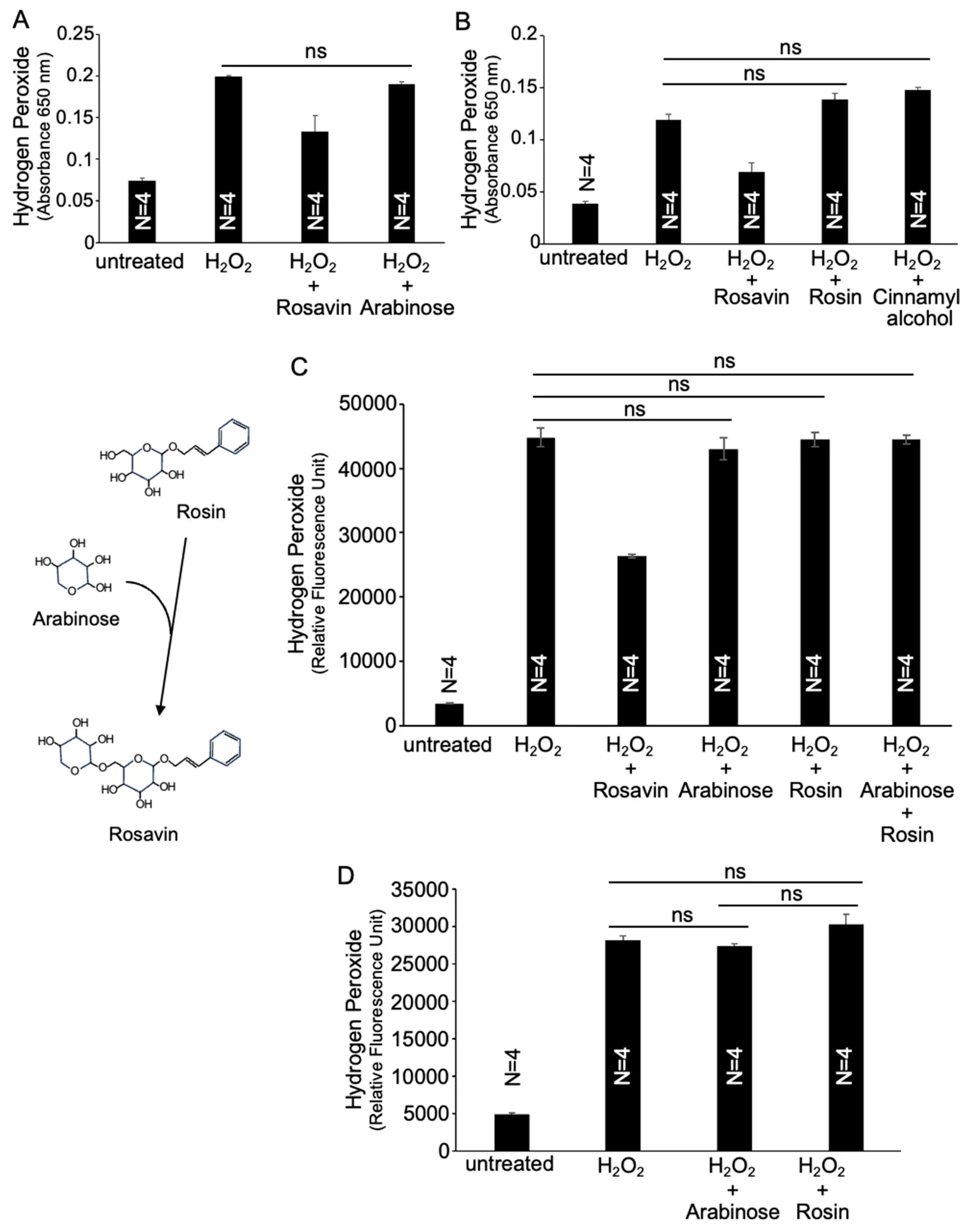
Structure–function relationship studies. (**A**,**B**) The Ampliate Colorimetric Hydrogen Peroxide Assay (AAT Bioquest) and (**C**,**D**) the EpiQuik In-Situ and Ex-Situ Hydrogen Peroxide Assay (EpigenTek) were used to determine the effects of rosavin, arabinose, and rosin on H_2_O_2_. The assay reactions contained a final concentration of 3 μM H_2_O_2_ and 150 μM rosavin, arabinose, rosin, and/or cinnamyl alcohol. (**D**) Effects of arabinose or rosin (750 μM) were also tested to ensure that the results showing the inability of these molecules to influence H_2_O_2_ are not due to insufficient amounts. The bar graphs represent the mean ± SEM. ns indicates no significant difference between groups. All the values without an “ns” designation are significantly different at *p* < 0.05 as determined by one-way ANOVA.

To investigate the mechanism underlying rosavin-mediated reduction of H_2_O_2_, we performed structure–function studies to determine which structural features are necessary for this activity. Rosavin is composed of arabinose, a monosaccharide, linked to rosin, a monoglucoside derivative of cinnamyl alcohol. Using the Amplite Colorimetric H_2_O_2_ Assay, we found that arabinose (Figure 5A), rosin (Figure 5B), and cinnamyl alcohol (Figure 5B) did not exhibit H_2_O_2_-reducing activity comparable to that of rosavin. Results from the EpiQuik Fluorescence H_2_O_2_ Assay confirmed these observations (Figure 5C). We further tested higher concentrations of arabinose and rosin (750 μM) and found that neither compound reduced H_2_O_2_ (Figure 5D). Additionally, the combination of arabinose and rosin together did not affect H_2_O_2_ levels (Figure 5C). Together, these findings suggest that the intact glycoside structure of rosavin defines its ability to inhibit H_2_O_2_.

### 3.3. Functional Roles of Rosavin Inhibition of H_2_O_2_

**Figure 6:**
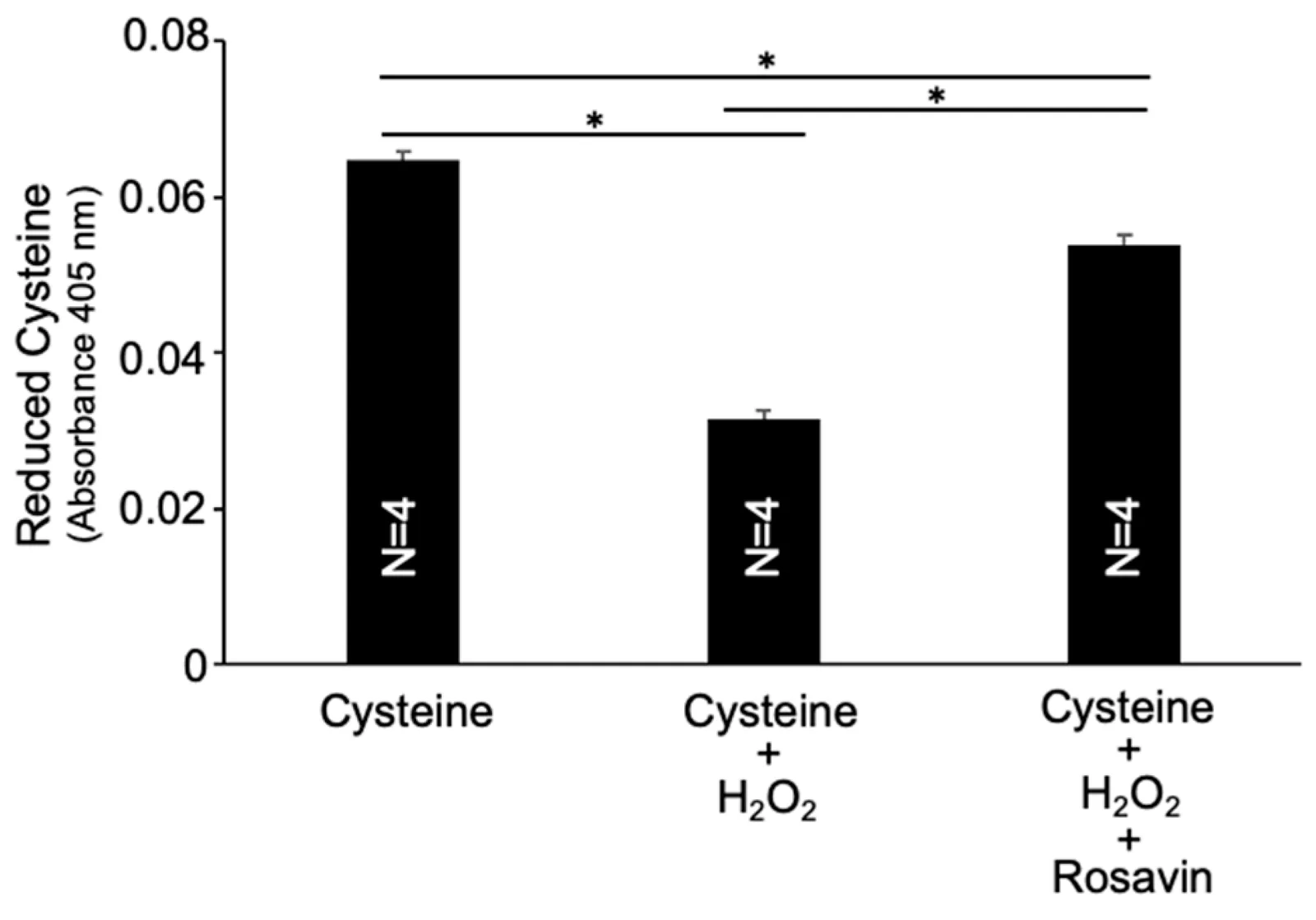
Rosavin inhibits H_2_O_2_-mediated oxidation of thiols. DTNB was used to monitor reduced thiol content. Reaction mixtures contained 0.5 mM DTNB, 1 mM L-cysteine, 25 μM H_2_O_2_, and 1 mM rosavin. The bar graph represents the mean ± SEM. * denotes groups that are significantly different from each other at *p* < 0.05 as determined by one-way ANOVA.

To assess the functional consequences of rosavin-mediated H_2_O_2_ inhibition, we first examined whether rosavin could attenuate H_2_O_2_-induced oxidation of thiol groups. 5,5′-Dithio-bis-(2-nitrobenzoic acid), DTNB, also known as Ellman’s Reagent, reacts with free sulfhydryl groups to produce 2-nitro-5-thiobenzoate (TNB), a yellow-colored product that can be detected by measuring absorbance at 412 nm [14]. As shown in Figure 6, L-cysteine containing a reduced sulfhydryl group reacted with DTNB to produce this signal, which was diminished upon oxidation by H_2_O_2_. Higher concentrations of H_2_O_2_ were required to produce a detectable effect in this assay. Addition of rosavin to this reaction effectively prevented H_2_O_2_-mediated oxidation of cysteine thiol groups, confirming rosavin’s capacity to preserve thiol redox status in the presence of H_2_O_2_.

**Figure 7:**
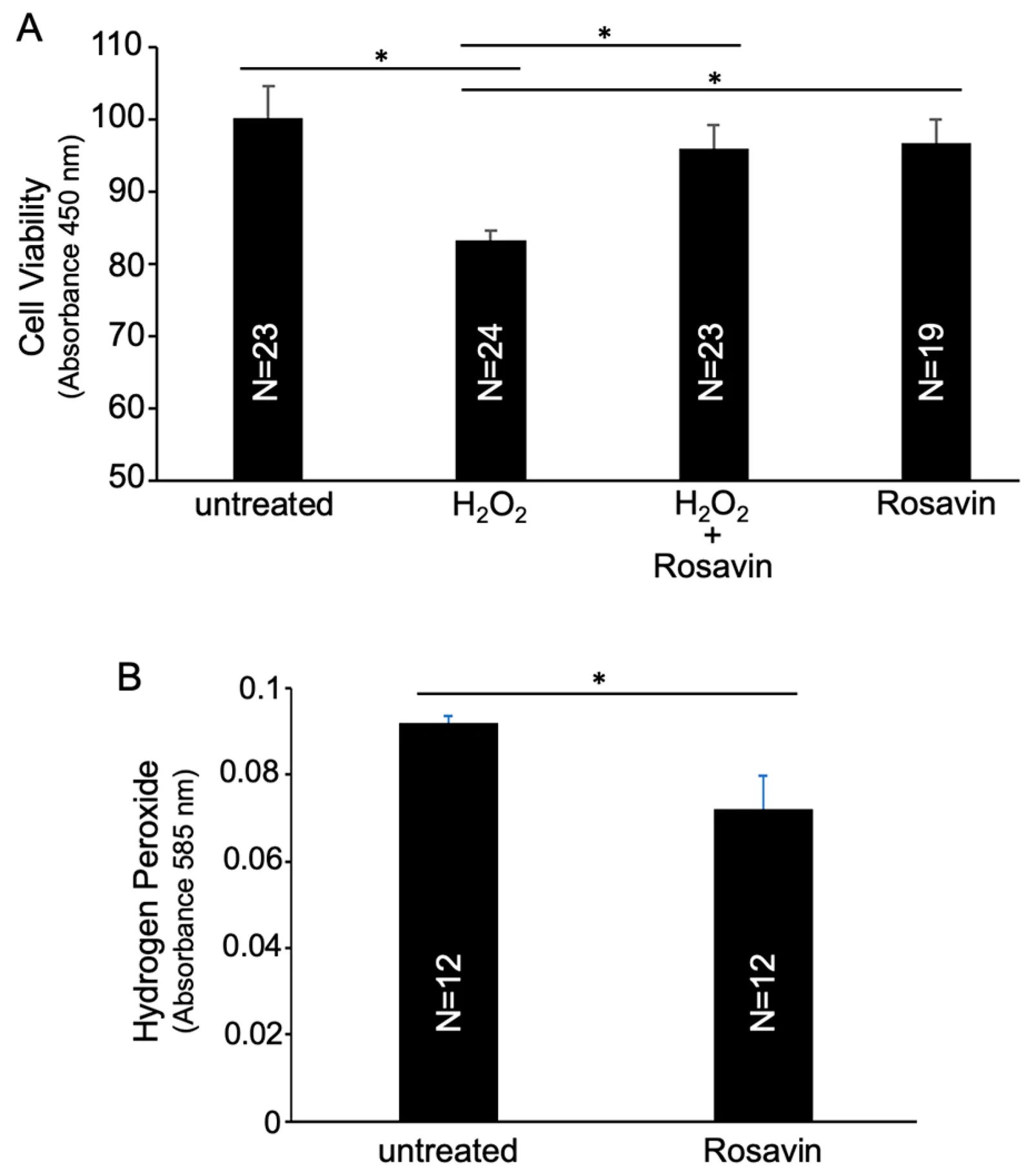
Effects of rosavin on H_2_O_2_-treated cultured cells. Cultured human pulmonary artery smooth muscle cells were treated with 1 μM H_2_O_2_ and 40 μM rosavin for 2 days. (**A**) Cell viability was determined using the CCK8 assay, demonstrating that rosavin attenuated the effects of H_2_O_2_ on cell viability. (**B**) H_2_O_2_ levels were measured in the culture medium using the QuantiChrom Peroxide Assay, showing that rosavin decreased H_2_O_2_ levels. The bar graphs represent the mean ± SEM of experimental replicates indicated in the graphs, which were obtained from two independent experiments for each assay. * denotes groups that are significantly different from each other at *p* < 0.05 as determined by (A) a one-way ANOVA or (B) Student’s *t*-test.

We next examined whether rosavin could mitigate H_2_O_2_-induced effects in cultured cells. H_2_O_2_ treatment of human cells decreased cell viability, as assessed by the CCK-8 assay, which measures cellular metabolic activity based on the reduction of the water-soluble tetrazolium salt (WST-8) by cellular dehydrogenases. Treatment with rosavin attenuated the H_2_O_2_-induced reduction in cell viability, supporting the cytoprotective role of rosavin during oxidative stress (Figure 7A). Rosavin alone did not affect cell viability compared with untreated controls (Figure 7A).

In this cell culture model, rosavin also decreased the levels of endogenous H_2_O_2_ in the culture medium (Figure 7B).

## 4. Discussion

The primary finding of the present study is that a key constituent of Rhodiola rosea extract, rosavin, reduces H_2_O_2_ levels in in vitro biochemical systems and cultured human cells. These findings provide mechanistic insights into the antioxidant and cytoprotective properties attributed to Rhodiola rosea extract and identify rosavin as a bioactive component whose effects depend on its intact chemical structure.

In this study, rosavin consistently attenuated H_2_O_2_ signals across several H_2_O_2_ detection assays. This pattern was further supported by the cysteine/DTNB assay, in which rosavin attenuated H_2_O_2_-mediated oxidation of cysteine thiol groups. In contrast, rosavin did not exhibit detectable reactivity toward superoxide in the xanthine/xanthine oxidase assay. Together, these findings suggest that rosavin exhibits measurable chemical reactivity with H_2_O_2_, but no detectable reactivity with superoxide under the conditions tested. Because rosavin requires a 30:1 to 40:1 molar excess to react with H_2_O_2_, it should be considered a weak H_2_O_2_ scavenger.

Rosavin contains a cinnamyl alcohol-derived phenylpropanoid backbone, consisting of an aromatic ring conjugated to an unsaturated three-carbon side chain [16]. This extended conjugation may permit partial electron delocalization and stabilization of radical or cationic intermediates that may form during redox interactions [17]. Although rosavin lacks the para-hydroxyl group typically associated with phenolic antioxidants, its conjugated phenylpropanoid scaffold itself may still support limited hydrogen atom transfer or electron transfer under certain chemical conditions [9]. These structural features plausibly explain rosavin’s ability to chemically reduce H_2_O_2_. While glycosidic substitution may reduce direct interaction of the aromatic system with oxidants, the underlying cinnamyl alcohol motif appears sufficient to support modest radical stabilization [18]. Nevertheless, our experimental results indicate that the intact rosavin structure is required to exert inhibitory activity toward H_2_O_2_ (Figure 5).

The degree of conjugation differs markedly between rosavin and salidroside. Salidroside’s tyrosol backbone consists of an aromatic ring attached to a saturated ethyl chain, lacking the extended π-system present in rosavin [2]. As a result, salidroside may have a reduced capacity to delocalize charge or stabilize radical intermediates, making electron or hydrogen donation less favorable [2]. This structural limitation may contribute to salidroside’s lack of detectable interaction with H_2_O_2_ in the chemical reactions examined here [11].

In summary, this study provides mechanistic insights into how *Rhodiola rosea* and its constituents may influence redox biology and highlights a structure-dependent role for rosavin in modulating H_2_O_2_. These findings contribute to a more detailed understanding of the functional properties of *Rhodiola*-derived compounds.

## Data Availability

The original contributions presented in this study are included in the article. Further inquiries can be directed to the corresponding author.

## Abbreviations

The following abbreviations are used in this manuscript:

ROS	Reactive oxygen species
H_2_O_2_	Hydrogen peroxide
DTNB	5,5′-Dithiobis-(2-nitrobenzoic acid)
TNB	2-nitro-5-thiobenzoate
